# Green Economy and Waste Management as Determinants of Modeling Green Capital of Districts in Poland in 2010–2020

**DOI:** 10.3390/ijerph20032112

**Published:** 2023-01-24

**Authors:** Piotr Misztal, Paweł Dziekański

**Affiliations:** Department of Economics and Finance, Jan Kochanowski University in Kielce, 25-369 Kielce, Poland

**Keywords:** zero waste, green economy, sustainable economy, synthetic measure, green capital, district, spatial differentiation

## Abstract

Waste management must comply with the principle of sustainable development. A green economy is one of the paths to sustainable development and response to environmental problems. Waste should be a raw material that can be reused, processed, or turned into energy. The aim of the article is to assess the relationship and present the concept of zero waste and green economy, and to show selected framework conditions for their development in the county. To study the formation of phenomena depending on the location of a given object in the socio-economic space, a synthetic measure, the similarity matrix, the Gini coefficient was used. The analysis took into account features describing the condition of the natural environment, links between the natural environment, economy and society, the quality of life of the population, economic policy, and instruments influencing the economy, society and the environment. Empirical data were obtained from the local database of the Central Statistical Office for the years 2010–2020. The obtained results indicate the existence of dependencies in the development of a green economy and waste management in the region. The synthetic measure of waste management is from 0.43 to 0.61 in 2010, from 0.39 to 0.55 in 2020; green economy from 0.42 to 0.55 in 2010 and from 0.43 to 0.56 in 2020. Waste management is based on multidimensional waste management, taking into account economic, ecological and social aspects. Economic activity is related to the degradation of the natural environment. The green economy should assume the elimination of waste and environmental threats and the preservation of their value. The green economy is becoming a factor inducing structural changes in the economy and social life, helping in the most effective, sustainable and efficient use of limited resources. In the literature on the subject, this type of research is carried out at the level of regions or voivodeships. The authors use their own sets of indicators or their own indicators aggregated according to the available data at the poviat level. The obtained results can be an important source of information for local government authorities about disproportions between units.

## 1. Introduction

Municipalities and districts are faced with the challenge of reacting to climate change, the scarcity of raw materials, the increasing amount of waste and a development process in which the integration of the three dimensions—social, economic and ecological—takes place [1]. Economic activity (in which three dimensions are integrated—social, economic and ecological) is based on the use of natural resources, capital and human resources. The existing development factors may also be a source of significant development constraints and even a limitation to further economic growth. Work, land and capital were also described as growth factors [2]. The green economy is a prerequisite for economic growth based on sustainable development, which can lead to human well-being and social justice, and significantly reduce environmental risks and bottlenecks. It can be defined as low-carbon, resource-efficient and inclusive [3]. The green economy is one of the paths to sustainable development. It is characterized by a stronger specification and operationalization of sustainable development. A multidimensional green economy is one that helps improve the quality of life and increase social equity while significantly reducing environmental risks. Increasing resource consumption and environmental costs overlap with increasing social inequality. Proper management of depleted natural resources requires treating waste as a valuable raw material that can be reused, processed or recovered as a last resort for energy recovery. The need to change the approach to waste management also stems from the need to reduce the valuable space required for their disposal, processing and storage [4]. Zero Waste is an approach that reorganizes the entire life cycle of resources so that all products can be reused. A minimal amount of waste goes to landfill or incinerators. The circular economy is the pursuit of a zero-waste economy. Its assumption is to extend the product life cycle. Keeping a given product in circulation for as long as possible reduces the mass of waste generated. It is about creating a closed loop that uses resources instead of creating waste. [5] Limited resources and unsustainable consumption can lead to ecological collapse and resource depletion. To be sustainable, it is imperative to achieve full resource efficiency to use fewer resources and produce less waste, but at the same time offering the same quality of service [6].

**Figure 1:**
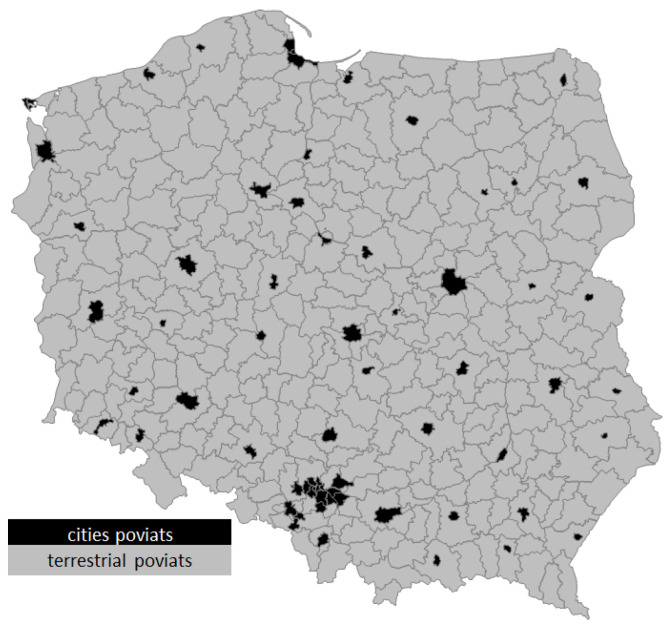
Research area—districts in Poland. Source: own study. Districts in Poland—380 (total); terrestrial districts—314; cities poviats—66.

Waste management (complex, interdisciplinary) should, in accordance with the principle of sustainable development, balance the needs of the economy, human comfort and the interests of the environment. The negative impact of waste on the natural environment leads to its deterioration. Man-made waste does not go away, it accumulates and produces pollutants that pollute soil, water and air. The need to change the approach to waste management also stems from the need to reduce the valuable space required for their disposal, processing and storage [7]. The tasks of districts include taking care of the process of sustainable development and improving the (green) quality of life, using local potential effectively, and giving residents and economic entities stability. The specified areas can be achieved while maintaining sustainable development (green development) in line with environmental protection and sustainable resource management [8]. Green growth is growth that is effective in using natural resources, minimizing pollution and its impact on the environment, the occurrence of natural hazards, green capital and environmental management in the process of local (or regional) economic change. Green growth means supporting economic development while ensuring that natural resources continue to provide the resources and environmental services on which our prosperity depends [9,10]. Green capital is defined by the authors as the sum of the values (tangible and intangible) of the green economy and waste management for the economy of the region. It is also a set of endogenous resources and environmental values that support the region’s pro-ecological development activities. Therefore, these are natural resources (wood, water, energy and mineral resources), biodiversity, ecosystems, ecological services (e.g., water and air filtration), and elements of green quality of life [11]. The authors see a research and literature gap in the area of green capital since this is not analyzed from an economic point of view, especially in the area of the rural districts (or municipalities). The aim of the article is to analyze and evaluate the spatial differentiation of green economy and waste management at the district level in Poland. Empirical data were collected in the spatial arrangement of the circles (Figure 1) in the location database of the Central Statistical Office for the years 2010–2020. The years of the study are the result of two application periods for EU funds and the availability of data contained in the CSO at the poviat level. Districts (poviat) are local self-governing communities, in other words, inhabitants and a given territory. It is an area of several to a dozen communes, or the area of a city with poviat rights (i.e., a commune with the status of a city, which has been granted poviat rights) [12]. The assessment was made using a synthetic measure. In pursuing the stated goal, the authors tried to answer the following questions: How spatially are green economy and waste management (green capital region) differentiated in terms of districts in Poland? Does the level of green economy and waste management depend on the level of (endogenous) variables of the development process? To what extent do the variables of the areas examined influence each other’s level?

## 2. Literature Review

Municipalities and districts shape the quality of life of the local community [13]. The converted activity is a multidimensional phenomenon that takes place in many parallel domains in economic, social and ecological areas [14]. The measures taken are intended to support the process of sustainable development and improve the quality of life, as well as strengthen skills and social cohesion, effective management of local resources (such as environment, people, infrastructure and financial resources) and a sense of stability for economic entities, reducing the insecurity of the business activity. The Quality of life is an important indicator of sustainable development. It is correlated with the economic situation of the region. The specified areas can be achieved while maintaining sustainable development in line with environmental protection and sustainable resource management [15].

The natural environment and the economy form a network of interrelationships and, when acting for the benefit of a particular community, are interdependent and should be viewed together. When considering the environmental conditions of regional development, attention should be paid to the concepts of independent regional development, green economy and zero waste. These concepts clearly indicate the need to consider environmental aspects in the process of regional development and to integrate the ecological aspect with the economic and social aspects [16].

The zero-waste concept is an answer to the dynamic increase in consumption and thus in waste. It shows both the avoidance of waste and the protection of raw materials, support for the cyclical use of raw materials, material reuse and the environmentally friendly recycling of residual materials [17]. Waste management is a complex, interdisciplinary concept that includes both the planning and implementation of projects and technologies as well as their control. Waste management can be viewed from a procedural and factual point of view. In waste management processes, waste prevention is preferred and the landfill is least desirable [18]. The item structure distinguishes types of waste, including municipal waste, generated in households, as well as in economic units, mainly service units, if they are similar in composition and type to those in households and do not contain hazardous waste [19]. The issue of waste management is highly complex and complicated at the same time due to its quantity, its diverse composition and its properties. This is due to the closely related aspects, namely the increase in the amount of waste, the increasing number of people, economic development, the nuisance and the threat to the environment and people from waste [20].

Waste management includes activities related to waste treatment (generation, management, recycling and disposal). Reuse, recycling and recovery become keywords of a new paradigm for sustainable development, innovation and competitiveness. Waste becomes a resource. Therefore, the new waste approach should positively influence a sustainable development process, the reduction in greenhouse gas emissions, a better environment and waste prevention, among other things. Intelligent waste management is an important tool for saving energy resources. Recycling and reuse contribute to a positive impact on the environmental footprint. The main goal of waste management is to create a cleaner and greener environment. A cleaner environment depends on reducing pollutants (water, air, soil, etc.). Rational waste management allows you to save energy through recycling or reuse practices [21].

The existing links in the economy, the existing networks of dependency and cooperation and the increasing unification of markets have become one of the causes of the current crisis (financial, economic, social and environmental) [22]. The increasing interdependence of the economies of the regions threatens, in the form of the transfer of negative phenomena, the economies connected in the closer cooperation networks and in the global system of flows (resources, production factors, capital, goods and services, vices, etc.) [23].

The Green Economy (GE) can be viewed as a way to drive economic growth and development while preventing environmental degradation, biodiversity loss and unsustainable use of natural resources. GE is a form of economic progress that promotes environmentally sustainable, low-carbon and inclusive development by ensuring environmental sustainability and maintaining the conditions for continued social progress [24,25]. A GE is an economy that combines economic growth with environmental responsibility. Economic development must be based on the principle of preserving synergy between social, economic and environmental aspects [26]. The added value of the GE is that the solutions created in it can be used in both modern and traditional industries [27].

Along with the rapid development of industrialization and urbanization, urban agglomerations have brought about accelerated spatial expansion, which has led to resource scarcity, ecological imbalance and environmental degradation. Its effect has been to weaken the regional green capital. Sustainable (green) regional development is hampered by the contradiction between resources, environment and socio-economic development. The natural environment is both the basis for commonly understood development and a barrier due to resource depletion. The finiteness of environmental resources compared to the unlimited needs of people requires a rational use of their resources. A green economy assumes that the economy is turning away from subordinating resources to satisfying the needs of the population towards adapting to environmental and globalization challenges [28]. A green economy can be analyzed sectorally (includes the following sectors: renewable energies, ecological building materials and energy-efficient construction, ecological transport, water and waste management) and spatially (spatial management, efforts to reduce environmental pollution) [29]. The concept of the green economy concerns, among other things, attributes of urban or regional resilience (adaptability, diversity, efficiency), resource conservation and minimizing the influence of external factors on intraregional processes [30].

A GE enables the harmonious management of local resources. A GE means restructuring economic activity and infrastructure to ensure higher returns on natural, human and economic capital. GE refers to the economic development issues related to sectors, regions and cities perceived as an element of the ecosystem. The essence of this approach is to create solutions that allow the economy to adapt more to the specificities of the environment [31]. The concept of a green economy includes three basic elements: eliminating environmental hazards and preserving their values, rational management of resources and natural resources, and social inclusion and economic efficiency. The concept of a green economy is complex, it includes all aspects of the economy (social, infrastructural, financial, etc.). The transition from a traditional economy to a green economy is a major change and will certainly affect almost all sectors of the economy (industry, trade, agriculture, tourism) [32].

The transition from a traditional brown economy to a green economy will be a gradual, complex process [33]. Focusing on sustainable development without environmental degradation, the green economy also promotes the concept of the triple bottom line, i.e., H. Profit, Society, Planet [34]. The benefits of a green economy (circular) include better resource efficiency, a reduced carbon footprint and reduced dependence on fossil resources, among others. This concept focuses on the idea of recycling, reusing and maintaining a sustainable production process. Sustainable and environmentally friendly disposal of waste is crucial to protect the environment and human health [35].

A GE is a way to gain and use resources. The associated structural changes in the economy are due to the emergence of new industries of waste recycling, zero-emission energy production, greenhouse gas emission absorption and green urban planning. These changes should be accompanied by a parallel increase in the quality of life of residents and sustainable development [36]. Increasing resource efficiency, promoting sustainable consumption and production, combating climate change, protecting biodiversity and the responsible use of natural resources and ecosystems are both a necessity and a driver for the transformation of the green economy [37]. The processes shown should also have a positive effect on the quality of life of the residents.

The green economy is becoming a factor of structural change in the economy and in social life. It contributes to a more effective and sustainable use of limited resources, which is part of the ZG influence on the development of territorial units. The orientation of sustainable development (or green economy) around the “three E’s” (environmental protection, economic growth and social justice) is also linked to considerations of quality of life [38]. It benefits the economy of the region socially, environmentally and economically as it provides better ways to use resources or eliminate environmental pollution and ecological growth of the region [39]. The green economy is interpreted as the 4Rs—i.e., reduction, reuse, recycling and recovery. These concern the reduction in resource consumption and the conservation of natural capital, the recovery of an energy resource (for example, the incineration of waste for heating) and consumption based on the continuous growth and increase in resource capacity by decoupling economic growth from environmental pressures [40].

Unlike the current model (brown economy), which is largely based on the use of fossil fuels and other non-renewable resources, the new model should draw on the experience of environmental economics and ensure an appropriate relationship between the economy and ecosystems [41]. Europe must strengthen the synergies between smart growth and green growth to face the challenges of climate change, the environment and energy as well as the scarcity of resources [42].

A green economy is a circular economy. It emphasizes the need to base the economy on renewable processes that promote biodiversity and bring benefits to people now and in the future [43]. The circular economy includes, among others: production and consumption, waste management and the secondary market of raw materials [44]. The circular economy model forces companies to reuse and repair, implementing traditional technologies through innovative methods. This approach advocates for the collection and recycling of residual waste to recover raw materials and/or convert them into usable heat, electricity and fuel [45].

The circular economy minimizes the use of non-renewable resources, the production of waste and pollution, and the reuse of materials. It is opposed to the approach of the linear economy, the operation of which can be represented as follows: acquiring raw materials, transforming them and then freely disposing of the product. Within the framework of the functioning of the regions, their territorial dimension refers to the concept of a collaborative economy. Subsequently, the unlimited consumption or accumulation of goods gives way to the sharing and exchange of goods [46].

## 3. Materials and Methods

The endogenous values of the region (its structure and its interconnections) are shaped by the functioning processes of the territorial units. The activities that are performed in multidimensional spaces are a combination of interconnected factors. Therefore, they must be analyzed and considered together as a set of interdependent elements (occurring on the same time horizon and interpenetrating). The development variables (financial situation, entrepreneurship, infrastructure, demography, labor market, natural environment, waste management) can be both internal and external from the point of view of the neighborhood and must represent a balanced whole.

The goal of multivariate comparative analysis is to determine a summary measure that allows the comparison of units (e.g., districts) described by many diagnostic variables. The following steps were used to analyze the spatial variation of green economy and waste management:

1. Determination of the set of diagnostic variables and the study area.
2. Reduction in the space of diagnostic variables (elimination of almost constant variables) verification in terms of statistics and content.
3. Normalization of variables—method of unitarization to zero and determination of the direction of preferences of variables in relation to the main criterion.
4. Determination of the value of the synthetic measure based on the formula selected for the aggregation of the diagnostic variables.
5. The linear arrangement of objects. Identification of typological classes for the whole range of variability of the synthetic measure, measures of descriptive statistics, and values of measures of similarity (similarity/dissimilarity matrix) were determined (Table 1, Table 3 and Table 4).

The basis of the analysis carried out was a set of homogeneous diagnostic variables which describe the elements of the environment, infrastructure, ecology, environmental efficiency of the production and life of the population and economic policies and their consequences. The initial diagnostic variables selected are somehow correlated with each other.

All the variables selected for the analysis are characterized by sufficient discriminant ability. The set of variables in the structure of synthetic measures (waste management, green economy) is presented in Table 2.

The choice of variables was largely determined by the availability of statistical data collected in the district system (some data were incomplete; data did not cover all districts). Some other variables have been omitted because their higher values do not necessarily indicate the level of socioeconomic development (e.g., water consumption per capita). Some other variables were removed because their higher values do not necessarily reflect levels of socio-economic development (e.g., population per library). Part of the data is incomplete; the data do not cover all districts.

From the set of diagnostic variables, those that did not provide any information on the phenomenon studied did not have the ability to differentiate (with too little variability) and showed a strong correlation with the variables that were deleted [55,56]. A high value of the correlation coefficient causes duplication of information on the analyzed phenomenon and can lead to erroneous conclusions [57]. It is believed that if the correlation coefficient is too high, a representative should be elected. The choice can be made on the basis of merit.

Variable selection was partly based on factor analysis in the Statistica program. The indicated method allows to convert the original set of objects into a set of groups, using orthogonal transformations of the original data matrix (e.g., factor analysis, principal component method) [58]. It allows us to reduce the number of variables analyzed and to transform the old system of variables into a new system made up of the main factors [59]. The main advantage of factor analysis is the ability to determine the number of hidden variables that adequately explain the interrelationship between many observable variables [60].

The selected primary diagnostic variables are obvious in relation to the main criterion in question: they are stimulating, and destimulating (Table 3) [61]. The nature of the selected variables can be controlled by determining the direction of the correlation of the variables with the decision variable (stimulants should be positively correlated with stimulants and destimulants negatively correlated with destimulants). For a stimulant, this direction should be positive and for a destimulant the direction should be negative [62].

Diagnostic variables usually have different titles and different fluctuation ranges, which makes direct comparison and addition impossible. In order to make the variables comparable, the null unitarization method was used, the purpose of which is to replace the different ranges of the variability of the individual variables with a constant range [63].

The synthetic measure (determined using the method Technique for Order Preference by Similarity to an Ideal Solution) makes it possible to evaluate the spatial differentiation of the units in the main criterion examined (Table 4). It allows a multidimensional vision of the phenomenon in the individual objects studied [64]. The first synthetic measure of development was developed by Z. Hellwig [65]. It provides the basis for the evaluation and comparison with the analyzed objects and allows to indicate the weakest and best functioning areas of the unit. It can be a useful tool to evaluate the correctness of past decisions and the effectiveness of previous regional management tools [66].

The TOPSIS method is a reference method in which two reference points are determined: the standard and the anti-standard [67]. A higher value of the measure indicates a better situation for an individual in the analyzed area [68].

**Table 4:** Stages 4, and 5 of building a synthetic measure.

Stage	Description of Stage	
stage 4	The *Technique for Order Preference by Similarity to an Ideal Solution (TOPSIS)* method is a reference method in which two reference points are determined—the standard and the anti-standard. Determining the Euclidean distances of objects from the pattern and anti-pattern, according to the formulas: (a) object distances from the pattern (=1):	
	$d_{i}^{+}=\sqrt{\frac{1}{n}\;\overset{m}{\underset{j=1}{\sum }}{\left(z_{ij}-z_{j}^{+}\right)}^{2}},$	(8)
	(b) distances of objects from the anti-pattern (=0):	
	$d_{i}^{-}=\sqrt{\frac{1}{n}\;\overset{m}{\underset{j=1}{\sum }}{\left(z_{ij}-z_{j}^{-}\right)}^{2}},$	(9)
	where *n*—denotes the number of variables forming the pattern or anti-pattern, $z_{ij}$—denotes the unitized value of the *j*-th feature for the tested unit (or the normalized value of the *j*-th variable of the object), $z_{j}^{+}/\;z_{j}^{-}$—denotes the template or anti-template object. Determining the synthetic measure (according to the TOPSIS method) according to the formula:	
	$q_{i}=\frac{d_{i}^{-}}{d_{i}^{-}+d_{i}^{+}},\;gdzie\;0\leq q_{i}\leq 1,\;i=1,\;2,\ldots ,\;n,$	(10)
	wherein: $q_{i}$ ∈ [0; 1]; $d_{i}^{-}—$means the distance of the object from the anti-pattern (from 0), $d_{i}^{+}$ means the distance of the object from the pattern (from 1). A higher value of the measure indicates a better situation of an individual in the analyzed area [52,53,54,63,65].	
stage 5	Division of the studied units into typological groups. The first, second and third quartiles were adopted as threshold values. The size of the synthetic measure in the first group means a better unit, and in the following groups—weaker units. The similarity matrix was determined in the PQStat program. The Euclidean distance is a metric and is given by the formula:	
	$d\left(A,B\right)=\sqrt{\left(x_{1A}-y_{1B}\right)+\left(x_{2A}-y_{2B}\right)+\ldots +\left(x_{nA}-y_{nB}\right)},$	(11)
	where *A* = (*x _a_*, *y _a_*), B = (*x _b_*, *y _b_*). Distance equal to 0 when they are identical. The farther away the objects are, the more dissimilar they are (=1). The similarity matrix was determined in the PQStat program. For the analysis and evaluation of the strength of the relationship between the variables and the synthetic measure of the studied areas, Pearson’s linear correlation coefficients (performed in the Grtel program) were used, expressed by the formula:	
	$r_{xy}=\frac{\sum_{i=1}^{n}\left(x_{i}-\overset{↼}{x}\right)\left(y_{i}-\overset{↼}{y}\right)}{\sqrt{\sum_{i=1}^{n}{\left(x_{i}-\overset{↼}{x}\right)}^{2}\sum_{i=1}^{n}{\left(y_{i}-\overset{↼}{y}\right)}^{2}}},$	(12)
	where, *r _xy_*—Pearson’s linear correlation coefficient, x and y are measurable statistical features *x* = (1,2,… n), *y* = (1,2,… n), and $\overset{↼}{x},\;\overset{↼}{y}$ are the arithmetic means of the features *x* and *y*. The Gini coefficient is a measure of the inequality of the distribution of the examined variable, it takes a value between 0 and 1 (the concentration coefficient was calculated in the Ststistica program). The Gini coefficient is expressed by the formula:	
	*G*(*y*) = $\frac{\sum_{i=1}^{n}\left(2i-n-1\right)y_{i}}{n^{2}\bar{y}},$	(13)
	where *y_i_* is the value of the *i*th observation and a $\bar{y}$ is the average value of all *y_i_* observations [69].	

Source: own study based on [52,53,54,63,65,69].

The result of the analysis was a synthetic measure to rank provinces according to their level of green management and waste management, taking into account long-term conditions.

The scatter plot and the pocket plot presented on the basis of the synthetic measure allowed us to show the differentiation of the units of the studied population and of the outliers (the graphs were created in the Statistica program).

The regression analysis was performed in the Gretl program.

Pearson’s linear correlation coefficients (run in the gretl program) were used to analyze and evaluate the strength of the relationship between the variables and the synthetic measure of the areas studied.

The Gini coefficient is a measure of the inequality of the distribution of the studied variable, it has a value between 0 and 1 (the concentration coefficient was calculated in the Statistica program). An index with a value of 0 indicates no inequality, while an index with a value of 1 means complete inequality [69].

## 4. Results

**Figure 2:**
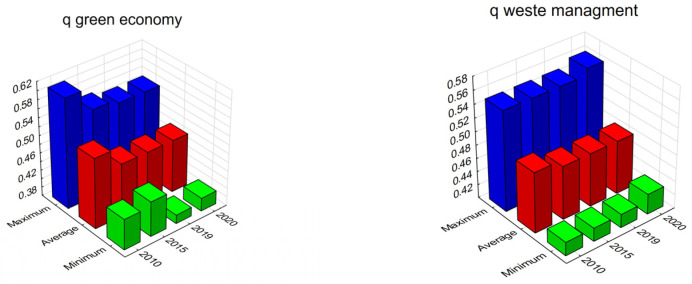
Synthetic measure waste management and green economy in districts in Poland (in 2010, 2015, 2019, and 2020). Source: own study.

The green economy and zero waste concept assume that the values of the natural environment are preserved for society. The indicated concept is determined by the conditions that ensure sustainable development. Waste is part of human life and economic activity. They become a source of pollution from the elements of the natural environment. The measure of synthetic waste management ranges from 0.43 to 0.61 in 2010, and from 0.39 to 0.55 in 2020. The average value of the measure remains at the level of 0.52 and 0.48. In the case of the green measure of economies, the values ranged from 0.42 to 0.55 in 2010 and from 0.43 to 0.56 in 2020 (with an average of 0.49 and 0.48). A higher value of the synthetic measure means a higher level of the phenomenon under consideration (see Figure 2).

**Figure 3:**
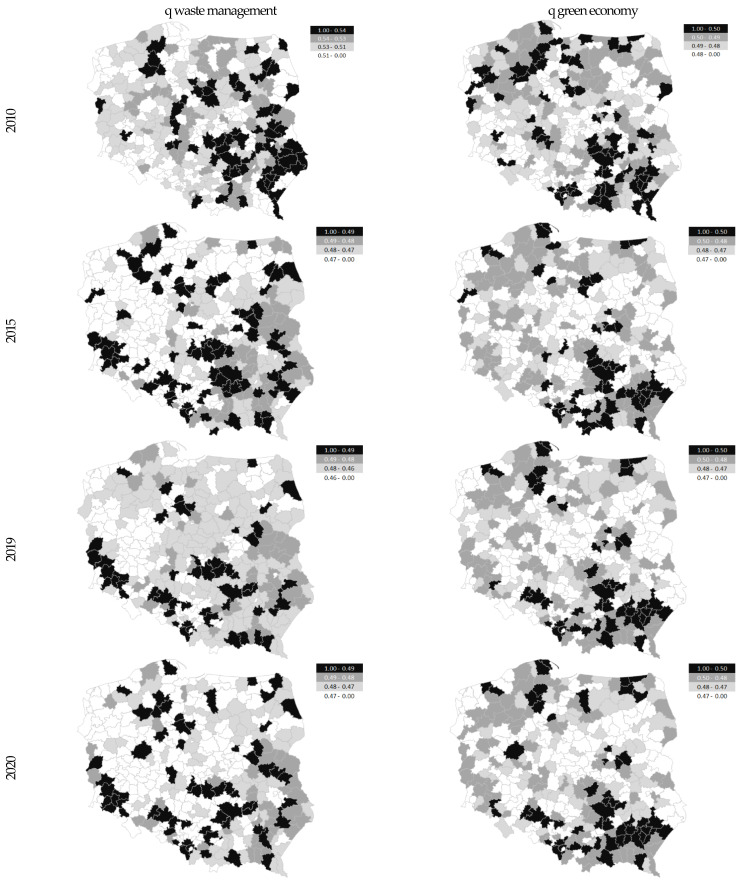
Spatial differentiation, waste management and green economy in districts in Poland (in 2010, 2015, 2019, and 2020). Source: own study based on the BDL CSO data.

The division of districts in Poland (into 2010, 2015, 209, and 2020) was made based on the value of quartiles. These were threshold values for the different typological groups. Figure 3 shows the distribution of neighborhoods according to the value of the synthetic measure, waste management and green economy. The black color indicates a group of neighborhoods, characterized by a better status in the main criterion examined, the lighter color was for the weaker units.

The similarity or dissimilarity of the units with respect to the main criterion examined (q waste management—the best unit—Nowosądecki district, the weakest unit Bydgoszcz, Polkowicki district; q green economy—Bielski, Kozieniecki and Wołowski) was expressed by the Euclidean Distance (Table 5). The distance is 0 if they are identical. The further away the objects are, the more dissimilar they are (=1). There is a greater differentiation with respect to the Green Economy measure and a lower level of waste management. The degree of differentiation was influenced by the function of the region (industrial, tourist, agricultural and tertiary).

Table 6 presents the statistical indicators of the synthetic measurement, waste management and green economy of the departments in the years 2010, 2015, 2019, and 2020. The results indicate a decrease in the spatial differentiation of the areas analyzed. All measures of central tendency (mean, median, quartile) are lower year over year (2010 to 2020). For volatility measures (range, standard deviation, coefficient of variation), we observe both the upside, the downside and the equilibrium. The higher the kurtosis, the more the community is concentrated around the mean (waste management for q), which results in a greater smoothness of the distribution curve. A decrease in the value of kurtoses (for q in the green economy) has the opposite effect, i.e., greater dispersion of values, low concentration and consequently a flattening of the curve of plenty.

**Figure 4:**
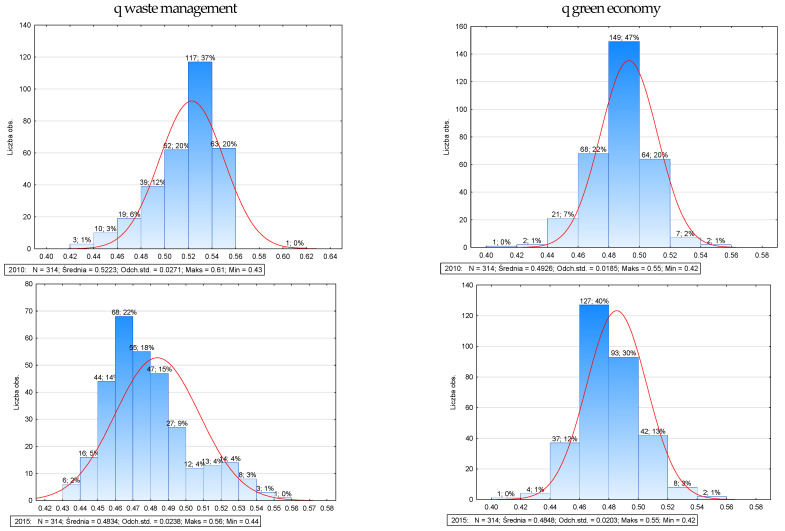

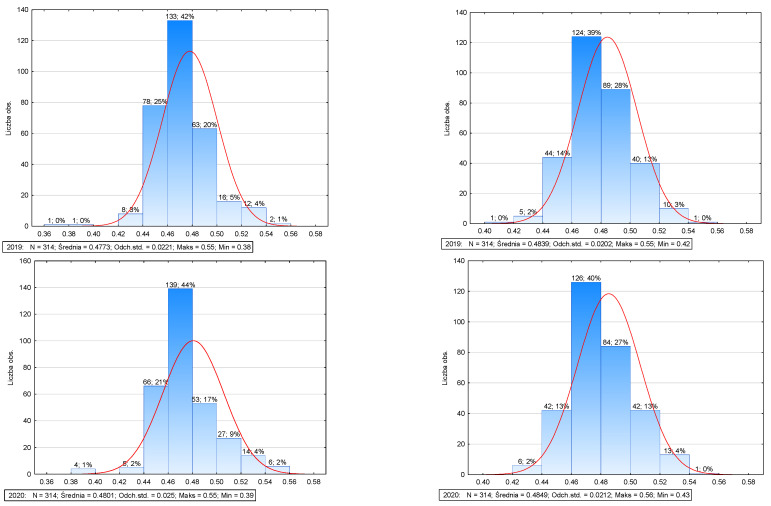
Distribution diagram of the synthetic measure, waste management and green economy in districts in Poland (in 2010, 2015, 2019, and 2020). Source: Own study.

Figure 4 shows the number of observations and the distribution pattern of the summary measure of waste management and green economy of neighborhoods in the years 2010, 2015, 2019, and 2020. In the analyzed measures of waste management and the green economy, we observe a left asymmetry (As < 0, in the year 2010) and on the right side (As > 0, in 2020). The left skew indicates that more units have values of these variables above their mean (the right skew is the reverse). The widest range was 0.52–0.54 (117.37%) for the Waste Management measure in 2010 and 0.46–0.48 (139.44%) for 2020, and 0.48–0.48 for the Green Economy measure; 0.50 (149.47%) and 0.46–0.48 (126, 40; this range is dominant).

**Figure 5:**
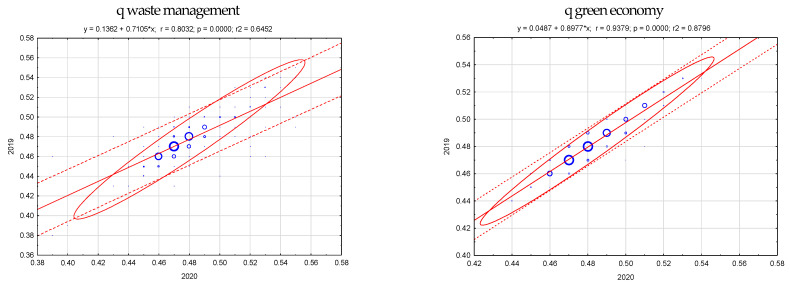

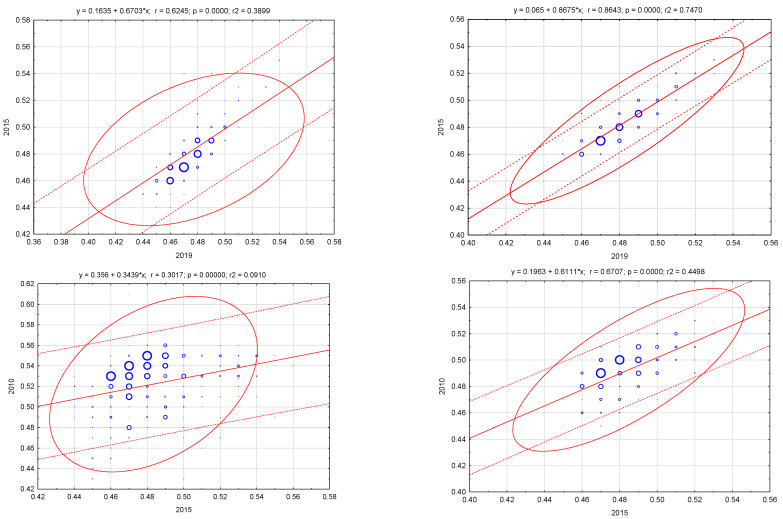
Diversity (spread) of the synthetic measure, waste management and green economy in districts in Poland (in 2010, 2015, 2019, and 2020). Source: Own study.

Scatterplot analysis shows what kind of relationship we are dealing with (positive or negative). This allows for the indication of groups of objects with similar values of the examined criterion, the indication of outliers (statistically distinct), and the indication of regularity (or irregularities) in the data set. The Pearson correlation coefficient between the value of the synthetic measure in the year-on-year report is shown in Figure 5. Higher correlation indicators in the year-on-year report are observed in the case of the green economy measure (the ratio of 2010 to 2015 and is 0.670; the ratio of 2020 to 2019 and is 0.937), lower for waste management (the ratio of 2010 to 2015 and is 0.301; the ratio of 2020 to 2019 and is 0.803).

**Figure 6:**
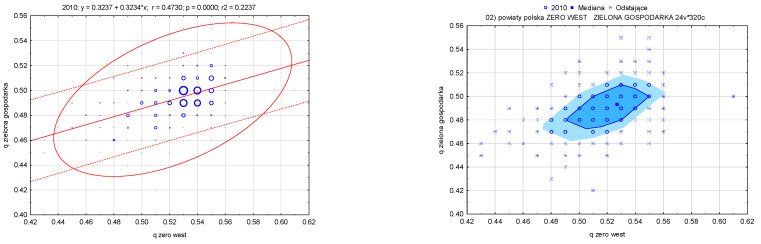

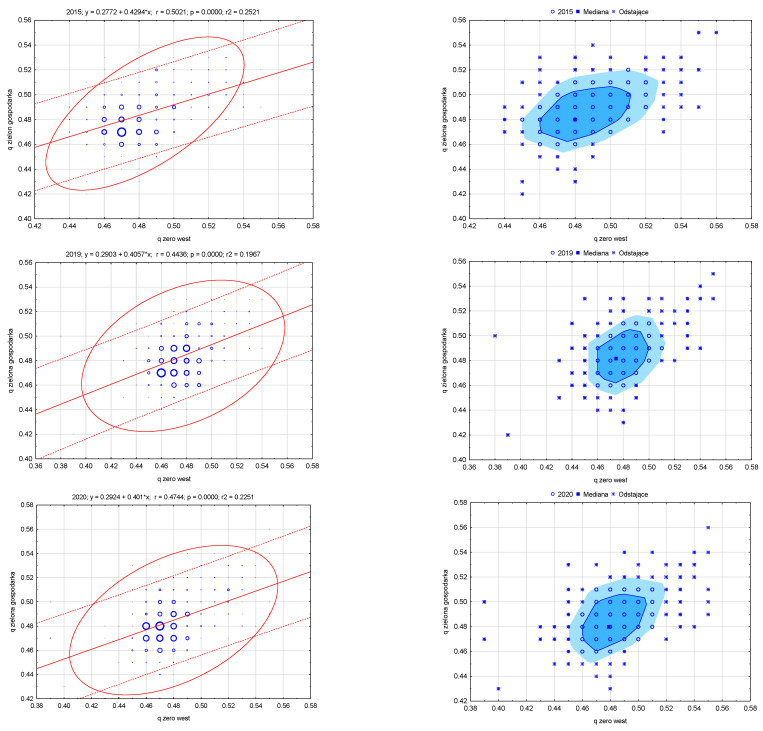
Relation of the synthetic measure, waste management and green economy in districts in Poland (in 2010, 2015, 2019, and 2020). Source: own study based on the BDL CSO data.

The Pearson correlation coefficient between the value of the measures analyzed in subsequent years was 0.473 and 0.474 for 2010 and 2020, respectively. The pocket graph shows statistically similar clusters of districts (including outliers, whose graphical form in the later years 2010 and 2015 as well as 2019 and 2020 indicate their slight differentiation) (Figure 6).

**Figure 7:**
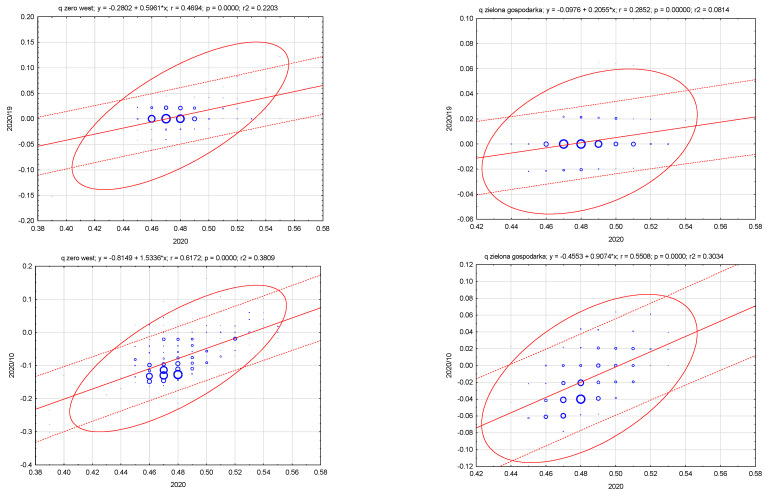
Relation of the synthetic measure, waste management and green economy and their changes in districts in Poland. Source: own study based on the BDL CSO data.

As Figure 7 shows, the summary metric of neighborhood waste management in 2020 versus 2020/2010 was subject to divergence (the correlation coefficient was 0.617), in the case of the green economy metric—0.550. In the case of the ratio between the measure in 2020 and the 2020/2019 ratio, it was, respectively, 0.469 (waste management) and 0.285 (green economy).

Waste must be recycled according to the principle of sustainable development or the concept of a green economy. Effective and truly sustainable waste management is an essential element of sustainable development. Such a system should consider both the quality of life of residents, the operation of businesses, and the environmental benefits that flow from effective waste management practices. Table 7 shows the Pearson correlation coefficient between the values of the waste management and green economy summary measure and the values of the diagnostic variables.

**Figure 8:**
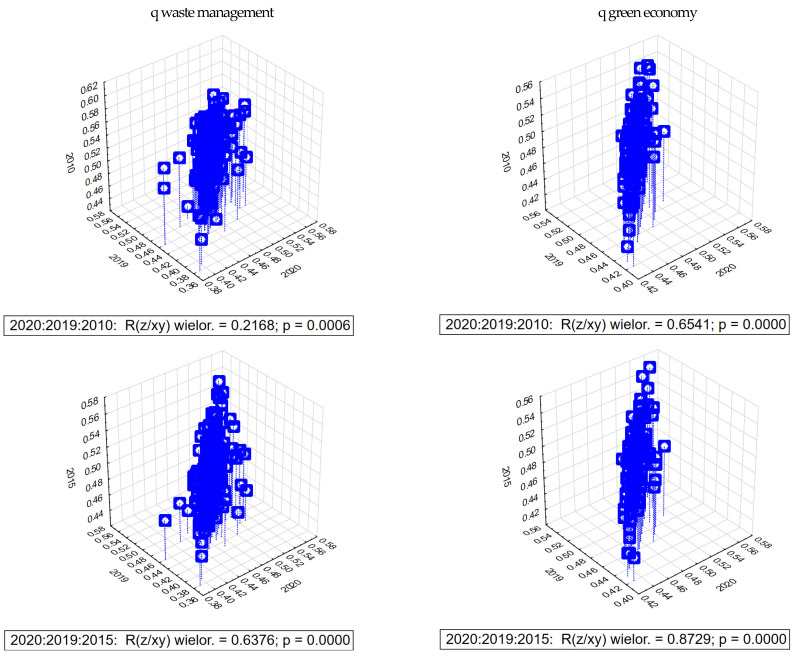
Interdependence of the synthetic measure of waste management and green economy of districts in relation to the years 2020-2019-2015 and 2020-2019-2010. Source: own elaboration based on BDL GUS data.

The visualization of the interdependence in three-dimensional space between the synthetic measure of sustainable development and waste management (in 2010, 2015, 2019, and 2020) is shown in Figure 8. In both cases analyzed, a process of divergence is observed (lower for the ratio of the measure on waste management 0.216, 0.637, and higher for the green economy 0.654, 0.872).

**Figure 9:**
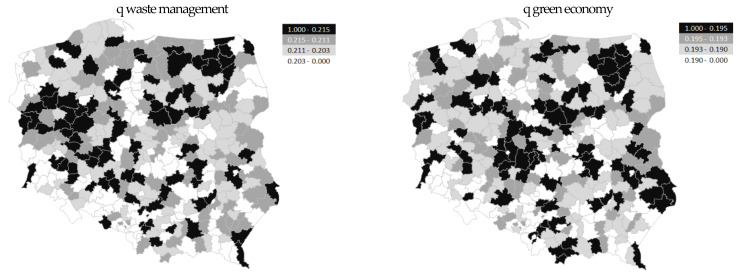
Concentration of the synthetic measure, waste management and green economy of districts (index Gini, for 2010–2020). Source: own study based on the BDL CSO data.

The measure for the distribution of the values of the synthetic measure waste management and green economy of neighborhoods for the years 2010–2020 has a value between 0 and 1. The higher the value of the indicator, the greater the concentration of the synthetic measure and the greatest diversification. Figure 9 shows the concentration of the phenomenon studied according to the synthetic measurement.

Taking into account the impact of waste management variables on green economy processes, the authors assess the impact of endogenous potentials of counties on the spatial variation of the synthetic measure of the green economy, and a linear regression model was estimated. Regression analysis of the synthetic measure of the green economy allows for explaining 0.81 variations of the variables. The values of the F statistic and the corresponding probability level *p* mean that all parameters are statistically significant (Table 8). The problem of counties in them is the paucity of endogenous factors that constitute a barrier to self-initiated development. 

## 5. Discussion

The green economy is a response to global environmental, economic and social problems. It must become a determinant of regional growth policy and international cooperation which essentially supports sustainable development [70]. A GE contributes to the improvement of human well-being and social equality while reducing ecological risks and consuming natural resources [71].

The concept of a green economy has been criticized for significantly overlapping with or attempting to replace sustainable development. The brand equates green economy with sustainable development because it cannot be precisely defined. Lorek and Spangenberg argue that the concept of a green economy does not support the original criteria of sustainable development. Addressing the artificial disconnect between the environment and the economy goes beyond sustainable development by providing a policy framework to achieve economic progress with reduced environmental impact [72]. Hickel shows the pitfalls of exponential growth, that is, the amount of steel, nonferrous metals, food, products, and energy we increasingly need. It points out that at the current rate of economic growth, a significant portion of new capacity from renewable energy sources will be used for growth itself and not to replace existing coal-fired power plants [73]. 

Sustainable development (or green economy) issues will have a huge impact on all aspects of human life in economic, social, environmental and political terms. The processes of structural transformation of economies are accompanied by growing inequalities in their development [74]. Both a green economy and sustainable development aim to improve the quality of life by ensuring the satisfaction of human needs and protecting the environment, natural and social resources and protecting the integrity of society [75]. Vukovic and others argue that green economy indicators have the properties of uncertainty and vagueness, which is why many authors have inadvertently used elements of fuzzy set theory to describe the research object. Finally, when developing the criteria to evaluate the green economy, researchers do not outline rules or strictly defined rules for their drafting [76]. 

The effectiveness of the green economy at the regional level comes with additional limitations compared to indicators at the national level. At the regional level, the direct transfer of indicators (both as a set of separate indicators and as part of an aggregated indicator) used at the national level often comes up against limitations in terms of data availability. Given this barrier, in studies devoted to the regional dimension of the green economy, the authors generally use their own sets of indicators or their own aggregated indicators according to the data available at the regional level (as indicated by the authors, the multidimensionality of the phenomenon poses a problem with regard to its unequivocal assessment, Table 2). 

The TOPSIS method used is part of the multi-criteria methods for assessing the spatial diversity of socio-economic phenomena. It makes it possible to classify regions, but also to assess progress towards a green economy. It was found that all Polish regions made progress in this respect, none of the examined regions had high values for all the variables included in the aggregate indicator (the maximum value was around 0.5 while the range of the indicator was [0,1]) [77]. The transition to a greener, resource-efficient and low-carbon economy is one of the pillars of the EU’s growth strategy and is at the heart of its implementation programs on issues such as the transition to a competitive economy carbon footprint by 2050, building a resource-efficient Europe and a circular economy. 

Analysis of waste management statistics and indicators shows a large variation between countries. Particularly important is the recycling rate, which is one of the benchmarks of the circular economy objectives. One of the first is Slovenia, which achieves a recycling rate on the level of highly developed countries, which is more than double that of Poland. Reuse and recycling must become part of everyday life for waste to practically cease to exist [78]. 

Green economies require environmentally sound and highly socially integrative economic growth both to protect and enhance the ecological environment and to make full use of natural resources to ensure the coordinated development of society, economy and life and environment. The development systems of the green economy are complex systems as they are influenced by economic, social, energy, environmental and technological factors. Therefore, when compiling the measurement indicators, care should be taken that each evaluation indicator effectively reflects the development status of the regional green economy, and takes into account the rationality of each indicator and its long-term usefulness [79].

Economic activity is based on the use of natural, capital and social resources. The change in approach to waste arises from the need to reduce the precious space required for disposal, treatment and storage [80]. In the area of waste management, particular attention is paid to avoiding waste generation and protecting raw materials, supporting the cyclical use of raw materials, reusing, recycling and recovering unavoidable waste, disposing of waste through safe landfills and using landfills as little as possible [81,82].

The study (conducted in three Swedish municipalities: Uppsala, Stockholm and Älvdalen) shows that reducing landfills in favor of more recycling of energy and materials leads to a lower environmental impact. When planning waste management, it is important to know that choosing a waste disposal method affects processes outside the waste management system, such as the generation of heat, electricity, automotive fuel, plastics, cardboard, and fertilizers. By recycling nutrients and materials, energy-intensive extraction and production of these raw materials are less necessary and the biogas from anaerobic digestion can be used as fuel for vehicles [83]. The ZW concept has been adopted by policymakers because it encourages sustainable production and consumption, optimal recycling and recovery of resources, and the reduction of mass incineration and landfill. However, waste management system specialists see and apply the concept of zero waste in different ways. For example, many studies have shown that LF targets are achieved using technologies to convert energy from waste, such as incineration. 

The circular economy has become one of the most important strategies for solving environmental problems. To enable a circular economy, organizations have started to respond to their ability to improve their sustainability. Recently, the economic and environmental performance has become a global requirement, and green finance and renewable energy have been identified as the key solutions put forward by researchers. Therefore, this study examines the potential link between the variables green finance, investment in renewable energy projects, economic performance and the environmental performance of OECD countries. Environmental performance is also a positive moderator in the relationship between green finance and economic performance and renewable energy investment and economic performance in OECD countries. The results of this study will help policymakers and practitioners in OECD countries and other similar regions of the world to properly plan their investments to achieve sustained economic growth and environmental sustainability [84].

The production and distribution of goods create waste and garbage that businesses must dispose of. Waste disposal is expensive because waste must be transported to a collection point. This is normally conducted in batches rather than piece by piece as waste generated continuously in the latter system would be very expensive. The location of the collection point is, therefore, crucial in determining the cost of transporting waste by companies [85]. The composition of the waste stream varies over time and space, with seasonal and long-term variations in the amount of different materials. Streamlining municipal waste management requires the establishment of management systems that ensure safe and systematic collection, cost-effective logistics and efficient/effective waste management services at all levels [86].

Significant differences in the generation of waste (including municipal waste) have been observed between EU countries. The amount of waste produced depended on economic development. The most effective tools for dealing with waste should be to improve reduce, reuse and recycle. Countries striving to minimize waste generation should also pay more attention to promoting sustainable consumption and production. The relationship between recycling behavior and waste generation was positive and statistically significant. Attitudes toward general waste management have a significant but negative impact on behaviors related to waste reduction and recycling. EU citizens are not aware of the link between waste reduction and resource efficiency [87].

Analysis of country data (Poland, Slovakia, Ukraine) showed a significant link between the basic elements of daily household functioning in terms of environmental protection through responsible consumption, reuse and recycling of certain products, packaging, materials and food and the Zero Waste Concept. Respondents consciously identify these activities as zero waste. The research results form the basis of public debate at the European and global levels in the field of creating legal regulations and educational programs in the context of waste management [88].

## 6. Conclusions

The green economy is a way of obtaining and using resources. It determines structural changes in the economy (e.g., waste recycling, emission-free energy production). The green economy includes green products and services, investments, green economic sectors, public procurement and jobs. The concept of the green economy becomes multidimensional and refers to the economic, social and ecological dimensions. It should have a positive impact on the quality of life of the inhabitants (e.g., by increasing resource efficiency, promoting sustainable consumption and production, combating climate change, protecting biodiversity, reducing pollution, and rational management of natural resources).

Waste management is important both from the point of view of the principle of sustainable development and the transformation process toward a green economy. Waste (its quantity and structure) has a negative impact on the natural environment. The problem of waste management is becoming a challenge for the modern economy (local, regional, and national). This is also due to the growing amount of waste, both in terms of production and consumption. In accordance with the principle of sustainable development, waste should be reused (recycled). Sustainable waste management requires a comprehensive procedure, taking into account economic, ecological and social aspects. Rational use of increasingly scarce natural resources requires treating waste as valuable raw materials. They can be reused, recycled, or as a last resort, used to produce energy.

The analysis took into account features describing the condition of the natural environment, links between the natural environment, economy and society, the quality of life of the population, economic policy, and instruments influencing the economy, society and the environment. The obtained results indicate the existence of dependencies (and connections) in the development of a green economy and waste management in the region. The synthetic measure of waste management is from 0.43 to 0.61 in 2010, from 0.39 to 0.55 in 2020; green economy from 0.42 to 0.55 in 2010 and from 0.43 to 0.56 in 2020.

Systematic research into the green economy and waste management should provide the government with the information it needs to evaluate and adjust policy. The increase or decrease in the synthetic measure should be treated as a way of evaluating the current management effects based on the main criterion. They can enable changes to be made toward optimizing the green economy and waste management. The obtained results are a source of information about differences between territorial units. For inter-regional comparisons, the proposed methodology must cover the same variables in the identified research areas.

The authors use their own sets of indicators or their own indicators aggregated according to the available data at the poviat level. The added value of the article is the systematization of knowledge in the aspect of green economy and waste management, as well as the analysis and assessment of their mutual dependencies in Polish poviats in the years 2010–2020. The indicated areas are usually analyzed separately. In the literature on the subject, this type of research is most often carried out at the level of regions or voivodeships.

With regard to the limitations related to the conducted research, the most important is the availability of data in the available statistics (e.g., Central Statistical Office in Poland), comparability of data, the evolution of regulations regarding the income system, tasks performed by local government units, budget reporting, changes in the administrative division, changes the socio-economic situation, and random events.

With regard to new research directions, the following can be specified: the selection of a larger number of diagnostic variables, the analysis over a longer period of time to identify trends of changes, and the selection of other methods to construct a collective measure. The results also indicate the need to analyze outliers and determine their impact on the situation of the study area.

## Figures and Tables

**Table 1:** Stages 1, and 2 of building a synthetic measure.

Stage	Description of Stage	
stage 1	A set of tested, multidimensional objects	
	Y = {Y _1_,… Y _n_}	(1)
	where n is the number of test objects. A set of diagnostic variables	
	X = {X _1_,… X _m_}	(2)
	where m is the number of studied variables, assuming that m ≥ n. Observation matrix (selected diagnostic variables) X_ij_:	
	$X_{\mathrm{ij}}=\left[\begin{array}{cccc} x_{11} & x_{12} & \ldots & x_{1m}\\ x_{21} & x_{22} & \ldots & x_{2m}\\ \ldots & \ldots & \ldots & \ldots \\ x_{n1} & x_{n2} & \ldots & x_{\mathrm{nm}} \end{array}\right]$,	(3)
	where: $X_{\mathrm{ij}}$—denotes the values of the j-th variable for the i-th object, matrix of dnaych objects, i—object number (i = 1, 2,..., n), j—variable number (j = 1, 2,..., m) [47].	
stage 2	Determination of the coefficient of variation, written by the formula:	
	$V_{i}=\frac{S_{i}}{\overset{-}{x}},$	(4)
	where, V _i_—coefficient of variation for the i-th variable, S _i_—standard deviation for the i-th variable, $\overset{-}{x}$ is the arithmetic mean of the i-th variable. From the set of variables, features meeting the inequality $\left|V_{i}\right|\leq {\;V}^{*}$ (critical value of the coefficient of variation = 0.10 were eliminated. Inverted matrix and correlation coefficient analysis, threshold value r* = 0.75 [48,49]. The selection of variables was also based on a factor analysis performed in the Statistca program.	

Source: own study based on [47,48,49,50,51,52,53,54].

**Table 2:** Describing variables—sustainable development, waste management.

Nr	Diagnostic Variables	Units	S/D
	waste management		
X1	Division 900—Municipal Management and Environmental Protection	pln/pc	S
X2	Total waste generated during the year per 1000 inhabitants	thousand t	D
X3	recovered per 1000 inhabitants	thousand t	S
X4	neutralized together per 1000 inhabitants	thousand t	S
X5	Waste previously stored (accumulated) in own facilities in total per 1 km^2^	thousand t	D
X6	share of recovered waste in the amount of waste generated during the year	%	S
X7	total per capita/Mixed waste collected during the year in total	kg	D
X8	Landfills/active landfills where municipal waste is neutralized—as of 31 December	pcs	D
X9	Non-reclaimed waste storage area per 1 km^2^	ha	D
X10	area of active landfills where municipal waste is neutralized—as of 31 December	ha	S
X11	wild landfill area per 100 km^2^ in total	pcs	D
X12	municipal waste collected during the liquidation of illegal landfills—during the year	vol	D
	Green economy		
X13	Expenses in Division 851—Health care	pln/pc	S
X14	Division 900—Municipal Management and Environmental Protection	pln/pc	S
X15	electricity consumption per capita/Electricity in households in cities	kWh	D
X16	electricity consumption per capita/Electricity in households by location of the recipient in the countryside	kWh	D
X17	waterworks Users of installations in% of the total population	%	S
X18	sewers	%	S
X19	Distribution network per 100 km^2^… water supply network	km	S
X20	sewage network	km	S
X21	gas network	km	S
X22	Yearly sales of heat energy by location, total residential buildings offices and institutions (per 1 inhabitant)	GJ	S
X23	The area of forest land in the total area	%	S
X24	water consumption per capita/Water consumption for the needs of the national economy and population during the year in total	m^3^	D
X25	share of industry in total water consumption	%	D
X26	total treated to total discharge Waste water treated during the year	%	S
X27	discharged per capita/Sewage treated during the year	dam^3^	D
X28	Population using sewage treatment plants in % of the total population	%	S
X29	share of recovered waste in the amount of waste generated during the year	%	S
X30	total per capita/Total mixed waste collected during the year	kg	D
X31	Municipal sewage treated per 100 km^2^	dam^3^	D
X32	Share of legally protected areas in the total area	%	S

S stimulant/D destimulant; The variables that repeat in both analyzed dimensions indicate the mutual dependence of the studied areas. Source: own study.

**Table 3:** The stages 3 of building a synthetic measure.

Stage	Description of Stage	
stage 3	The normalization of diagnostic variables was performed depending on their types of variables, X j ∈ S according to the formula:	
	$Z_{\mathrm{ij}}=\frac{x_{\mathrm{ij}}-\min_{i}x_{\mathrm{ij}}}{\max_{i}x_{\mathrm{ij}}-\min_{i}x_{\mathrm{ij}}}$, Z _ij_ = 0 ⇔ x _ij_ = min _i_ x _ij_; Z _ij_ = 1 ⇔ x _ij_ = max _i_ x _ij_.	(5)
	for the variable X j ∈ D,	
	$Z_{\mathrm{ij}}=\frac{\max_{i}x_{\mathrm{ij}}-x_{\mathrm{ij}}}{\max_{i}x_{\mathrm{ij}}-\min_{i}x_{\mathrm{ij}}}$, Z _ij_ =0 ⇔ x _ij_ = max _i_ x _ij_; Z _ij_ =1 ⇔ x _ij_ = min _i_ x _ij_,	(6)
	where: Z _ij_ ∈ [0; 1], max _i_ x _ij_ ≠ min _i_ x _ij_, max _i_ x _ij_ > min _i_ x _ij_, S-stimulant, D-destimulant, i = 1, 2… n (number of selected variables for analysis); j = 1, 2… m (number of random values of the variable), max_xij_—the maximum value of the j-th variable, min_xij_—the minimum value of the j-th variable, x _ij_—means the value of the j-th variable for the th object [48,50]. Value matrix of unitary features $Z_{\mathrm{ij}}$:	
	$Z_{\mathrm{ij}}=\left[\begin{array}{cccc} z_{11} & z_{12} & \ldots & z_{1m}\\ z_{21} & z_{22} & \ldots & z_{2m}\\ \ldots & \ldots & \ldots & \ldots \\ z_{n1} & z_{n2} & \ldots & z_{\mathrm{nm}} \end{array}\right]$,	(7)
	where Z_ij_ ∈ { S} ∪ {D}—unitized value of j-th variables for i-th object; i = 1,..., m, j = 1,..., k, are the normalized values of the jth diagnostic variable for this object.	

Source: own study based on [48,49,50,51,52,53,54].

**Table 5:** Distance between the best and the weakest unit (according to the similarity matrix) for the synthetic measure waste management and green economy of districts.

q Green Economy				q Waste Management			
2010–2015							
	Bielsko district	Kozienice district	Wolow district		Nowy Sącz district	Bydgoszcz district	Polkowice district
Bielsko district	0	0.16	0.13	Nowy Sącz district	0	0.08	0.04
Kozienice district	0.16	0	0.06	Bydgoszcz district	0.08	0	0.06
Wolow district	0.13	0.06	0	Polkowice district	0.04	0.06	0
2015–2019							
Bielsko district	0	0.17	0.18	Nowosądecki district	0	0.11	0.17
Kozienice district	0.17	0	0.01	Bydgoszcz district	0.11	0	0.09
Wołowski district	0.18	0.01	0	Polkowice district	0.17	0.09	0
2019–2020							
Bielsko district	0	0.18	0.18	Nowosądecki district	0	0.18	0.23
Kozienice district	0.18	0	0.01	Bydgoszcz district	0.18	0	0.08
Wołowski district	0.18	0.01	0	Polkowice district	0.23	0.08	0

Source: own study.

**Table 6:** Statistical characteristics of the synthetic measure waste management and green economy in districts in Poland (in 2010, 2015, 2019, and 2020).

	q Green Economy				q Waste Management			
	2010	2015	2019	2020	2010	2015	2019	2020
Mean	0.52	0.48	0.48	0.48	0.49	0.48	0.48	0.48
Median	0.53	0.48	0.48	0.48	0.49	0.48	0.48	0.48
Minimum	0.43	0.44	0.38	0.39	0.42	0.42	0.42	0.43
Maximum	0.61	0.56	0.55	0.55	0.55	0.55	0.55	0.56
Lower (Quartile)	0.51	0.47	0.46	0.47	0.48	0.47	0.47	0.47
Upper (Quart.)	0.54	0.49	0.49	0.49	0.5	0.5	0.5	0.5
Gap	0.18	0.12	0.17	0.16	0.13	0.13	0.13	0.13
Quartile. (Gap)	0.03	0.02	0.03	0.02	0.02	0.03	0.03	0.03
SD	0.03	0.02	0.02	0.03	0.02	0.02	0.02	0.02
Coefficient of change	5.18	4.91	4.64	5.21	3.76	4.19	4.18	4.36
Skewness	−0.87	0.81	0.28	0.32	-0.32	0.37	0.31	0.41
Kurtosis	0.85	0.33	2.33	1.86	1.04	0.39	0.26	0.26

Source: own elaboration based on BDL GUS data.

**Table 7:** Coefficients of correlation between the value of the synthetic measure, waste management, green economy and diagnostic variables of their structure for districts in 2020 and 2020.

Diagnostic Variable/Specification	q Green Economy	q Waste Management
q waste management	0.474	1.000
recovered per 1 km^2^	0.188	0.230
share of recovered waste in the amount of waste generated during the year	0.496	0.757
total per capita/Total mixed waste collected during the year	−0.087	−0.425
area of illegal dumps per 100 km^2^ of total area	−0.230	−0.287
q green economy	1.000	0.474
expenditure in chapter 90003—Clearing towns and villages	0.081	−0.243
expenses in chapter 90004—Maintenance of green areas in cities and communes	0.029	−0.170
expenses in chapter 90015—Lighting of streets, squares and roads	−0.112	−0.120
electricity consumption per capita/Electricity in households by location of the recipient in the countryside	−0.196	−0.203
waterworks Users of installations in% of the total population	−0.039	−0.162
sewers	0.396	−0.146
gas	0.381	−0.094
Distribution network per 100 km^2^… water supply network	0.254	−0.005
sewage network	0.543	0.012
gas network	0.470	0.018
The area of forest land in the total area	0.104	0.198
water consumption per capita/Water consumption for the needs of the national economy and population during the year in total	−0.208	−0.024
industry share in total water consumption	−0.269	−0.089
total discharged sewage treated during the year/per 1 km^2^	0.330	−0.133
Population using sewage treatment plants as a percentage of the total population	0.413	−0.133
share of recovered waste in the amount of waste generated during the year	0.496	0.757
total per capita/Total mixed waste collected during the year	−0.087	−0.425
Municipal sewage treated per 100 km^2^	0.330	−0.133
existing area—as of 31 December	−0.248	−0.249

Linear correlation coefficients for observations from sample 1–314/ Critical value (with two-tailed 5% critical area) = 0.1107 for n = 314. Source; developed on the basis of BDL GUS data.

**Table 8:** Results of regression analysis for the synthetic measure green economy.

Arithmetic mean of the dependent variable	0.484904	Standard deviation of dependent variable	0.021153
Sum of squares residuals	0.026585	Residual standard error	0.009367
Coefficient of determination R-square	0.810173	Adjusted R-square	0.803908
F(10, 303)	129.3191	p-values for F-test	4.88 × 10³
Logarithm of credibility	1026.613	Inrom. Crit. Akaike’a	−2031.226
Crit. Bayes. Schwarza	−1989.982	Crit. Hannana-Quinna	−2014.746

Source: own study based on the BDL CSO data.

## Data Availability

Data sharing not applicable.
